# Supplementary material for: The evolution of antibiotic resistance is associated with collateral drug phenotypes in Mycobacterium tuberculosis
Source: Nat Commun. 2023 Mar 18;14:1517. doi: 10.1038/s41467-023-37184-7 (PMC10024696; doi:10.1038/s41467-023-37184-7)
Supplement: Supplementary file 1 — Supplementary information [file 41467_2023_37184_MOESM1_ESM.pdf]

Supplementary Information: The evolution of antibiotic resistance is associated with collateral drug phenotypes in *Mycobacterium tuberculosis*

Natalie J.E. Waller[1,2], Chen-Yi Cheung[1], Gregory M. Cook[1,2] and Matthew B. McNeil[1,2#]

1: Department of Microbiology and Immunology, University of Otago, New Zealand.

2: Maurice Wilkins Centre for Molecular Biodiscovery, University of Auckland, New Zealand.

#Corresponding Author

matthew.mcneil@otago.ac.nz

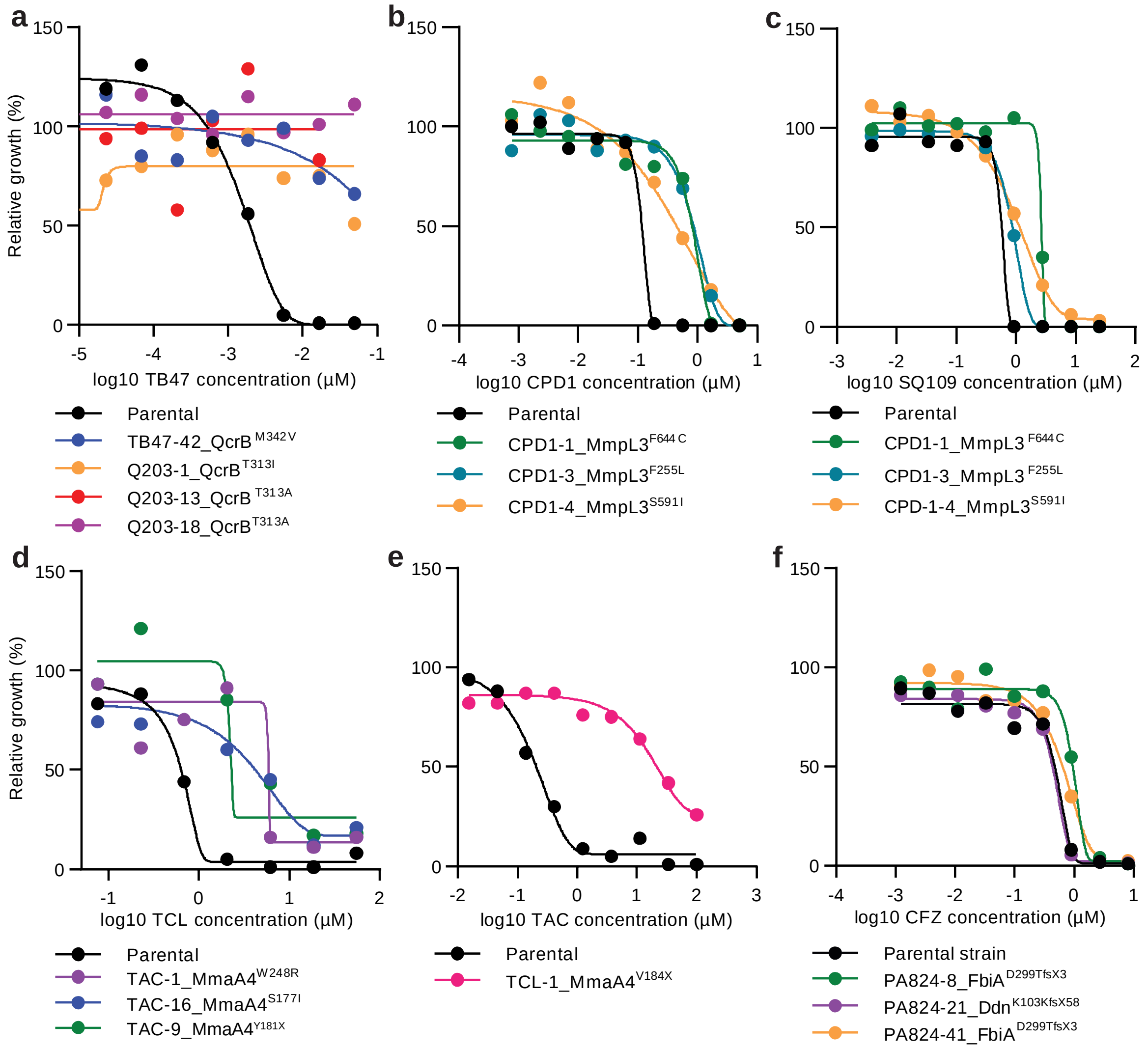

Supplementary Figure 1: Cross-resistance in drug-resistant variants of *M. tuberculosis*. (a-f) examples of cross-resistance seen in dose-response curves for selected drug-resistant variants and the drug-susceptible parent against selected antibiotics. Strain names and candidate mutations are listed below each dose-response. Dose-response curves are the results of a single biological replicate from a representative experiment (n>2 independent experiments).

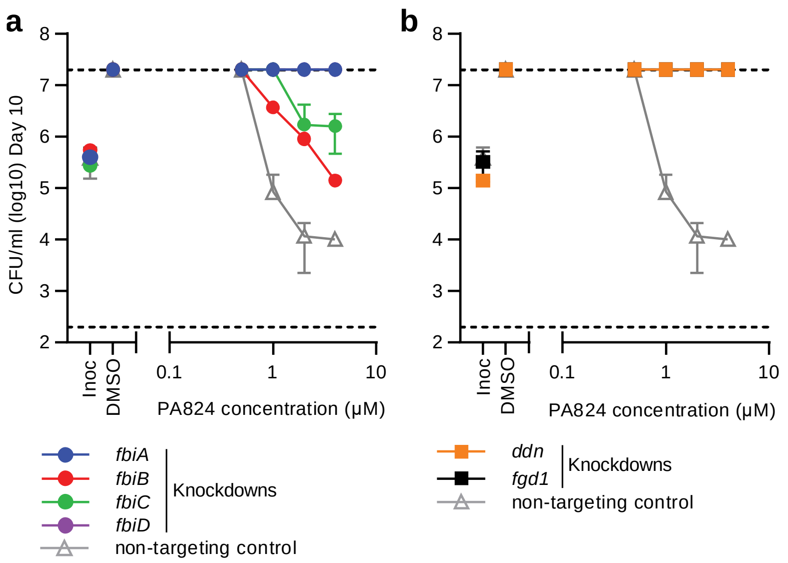

Supplementary Figure 2: Minimum bactericidal concentration assays for CRISPRi knockdown strains against PA824. (a+b) Minimum bactericidal concentration assays for CRISPRi knockdown and non-targeting strains against PA824. CFUs were determined at day 0 and at day 10. Inoc = starting inoculum on day 0, DMSO = solvent control. Data is the mean ± range of biological duplicates from a representative experiment (n=2 independent experiments). Dashed lines represent the upper and lower limits of detection.

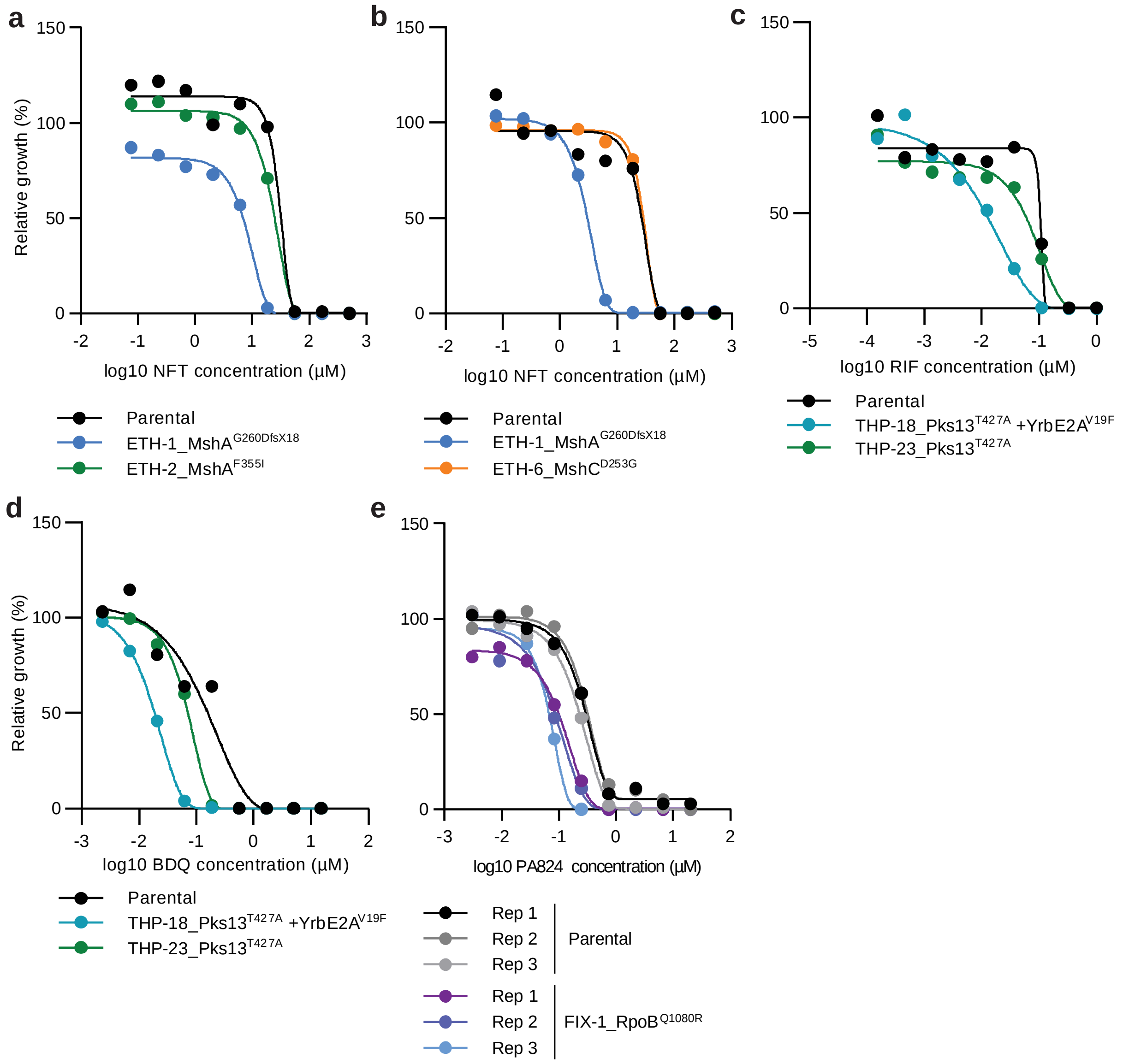

Supplementary Figure 3: Collateral sensitivity in drug-resistant variants of *M. tuberculosis*. (a-e) examples of collateral sensitivity seen in dose-response curves for selected drug-resistant variants and the drug-susceptible parent against selected antibiotics. Strain names and candidate mutations are listed below each dose-response. (a-d) Points on dose-response curves are the mean of biological duplicates from a representative experiment (n>2 independent experiments). e = a representative experiment with 3 technical replicates.

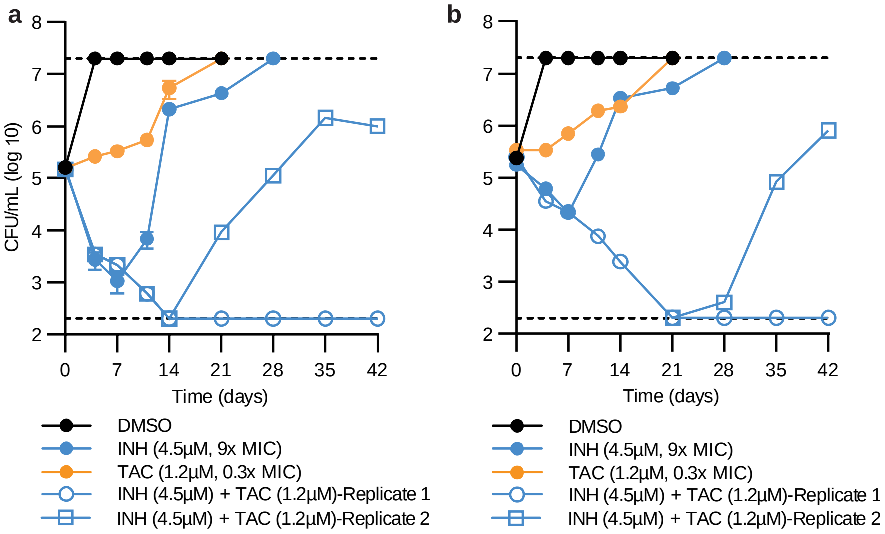

Supplementary Figure 4: Additional replicate experiments of INH + TAC combination studies to show variation in the time taken for resistance to arise. (a+b) Drug-susceptible *M. tuberculosis* was incubated with either INH at 9x the MIC, below MIC concentrations (i.e., 0.3x MIC of the drug-susceptible parent) of TAC, or a combination of INH (9x MIC) and 0.3x MIC of TAC. CFUs were determined on the stated days. Data is the mean ± range of biological duplicates, with each graph representing an independent experiment. Dashed lines represent the upper and lower limits of detection.

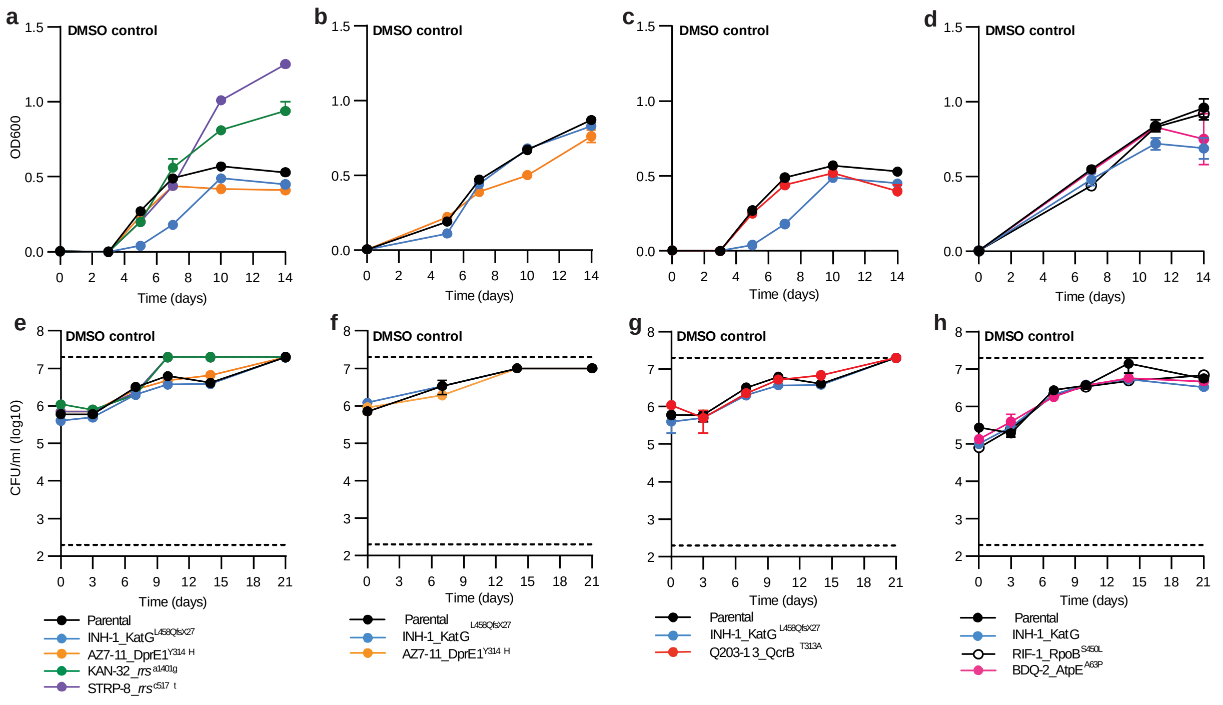

Supplementary Figure 5: DMSO control curves for relevant assays. (a-d) Solvent control OD_{600} curves for Figure 3e-h. (e-h) Solvent control CFU curves for Figure 4e-h. (a-h) Data is presented as the mean ± range of biological duplicates from a representative experiment (n>2 independent experiments). Dashed lines represent the upper and lower limits of detection.

**Supplementary Table 1: Expanded information for compounds used in this study**					
**Antibiotic**	**Abbreviation**	**Target**	**Supplier**	**Catalogue/SKU number**	**Liquid MIC against *M. tuberculosis* mc^{2}6206 susceptible parent strain (μM)**
Rifampicin	RIF	Transcription	Sigma-Aldrich	R3501	0.1
Fidaxomicin	FIX	Transcription	Sigma-Aldrich	SML1750	4
Linezolid	LZD	Translation	Sigma-Aldrich	PZ0014	3
Kanamycin	KAN	Translation	Sigma-Aldrich	60615	4
Streptomycin	STRP	Translation	Sigma-Aldrich	S9137	0.15
Capreomycin	CAP	Translation	Selleck Chemicals	S4234	1.1
Levofloxacin	LEV	DNA gyrase	Sigma-Aldrich	28266	0.8
Bedaquiline	BDQ	Bioenergetics	Toronto research chemicals	HY-14881	1
Clofazimine	CFZ	Bioenergetics	Sigma-Aldrich	C8895	0.5
Q203	Q203	Bioenergetics	Courtesy of Kevin Pethe		0.01
TB-47	TB47	Bioenergetics	Courtesy of Xiaoyun Lu		0.003
Pretomanid	PA824	Bioenergetics / cell wall	Sigma-Aldrich	SML1290	0.8
Isoniazid	INH	Cell wall	Sigma-Aldrich	I3377	0.5
Ethionamide	ETH	Cell wall	Sigma-Aldrich	E6005	10
Ethambutol	EMB	Cell wall	Sigma-Aldrich	E4630	3
PBTZ-169	PBTZ	Cell wall	Cayman Chemical	22202	0.001
AZ7371	AZ7	Cell wall	Cayman Chemical	19310	1
SQ109	SQ109	Cell wall	Sigma-Aldrich	SML1309	0.8
Thioacetazone	TAC	Cell wall	Santa Cruz Biotechnology	sc-358574	4
Thiocarlide	TCL	Cell wall	Cayman Chemical	10006976	5
Thiophene-2	THP	Cell wall	Sigma-Aldrich	SML1120	1.5
Compound-1	CPD1	Cell wall	Courtesy of Tanya Parish (Molecule #4*)		0.1
Tunicamycin	TUN	Cell wall	abcam	ab120296	1.5
Nitrofurantoin	NFT	Undefined	Sigma-Aldrich	N7878	50
* DOI: 10.1021/acsomega.0c05589					

**Supplementary Table 2: sgRNA targeting PA824 resistance genes in *M. tuberculosis***						
**Target**	**sgRNA name**	**PAM sequence (5'-3' template strand)**	**PAM score***	**Targeted sequence (coding strand 5'-3')**	**Forward oligo**	**Reverse oligo**
fbiA / Rv3261	FbiA_a	GCAGAAT	2	CCACTCGGACGCCGACCACC	GGGAGGTGGTCGGCGTCCGAGTGG	AAACCCACTCGGACGCCGACCACC
fbiB / Rv3262	FbiB_a	CCAGAAC	5	CTCGGGACCGCCGAAGC	GGGAGCTTCGGCGGTCCCGAG	AAACCTCGGGACCGCCGAAGC
fbiC / Rv1173	FbiC_a	CGAGGAT	9	ACGTTGCCCGCCGAGGTGCC	GGGAGGCACCTCGGCGGGCAACGT	AAACACGTTGCCCGCCGAGGTGCC
fbiD / Rv2983	FbiD_a	CGAGCAT	7	TCGACACGTTGACCGCCGCGGC	GGGAGCCGCGGCGGTCAACGTGTCGA	AAACTCGACACGTTGACCGCCGCGGC
ddn / Rv3547	Ddn_a	AAAGAAG	1	ATCAAGTGGATGTCACGGAT	GGGAATCCGTGACATCCACTTGAT	AAACATCAAGTGGATGTCACGGAT
fgd1 / Rv0407	Fgd1_a	CGAGAAC	5	CTGTCCTGGATGACCGCTGT	GGGAACAGCGGTCATCCAGGACAG	AAACCTGTCCTGGATGACCGCTGT
* PAM score ranked out of 15 possible permissible sequences (DOI:10.1038/nmicrobiol.2016.274)						
