# Supplementary material for: The evolution of antibiotic resistance is associated with collateral drug phenotypes in Mycobacterium tuberculosis
Source: Nat Commun. 2023 Mar 18;14:1517. doi: 10.1038/s41467-023-37184-7 (PMC10024696; doi:10.1038/s41467-023-37184-7)
Supplement: Supplementary file 3 — Description of Additional Supplementary Files [file 41467_2023_37184_MOESM3_ESM.pdf]

Description of additional supplementary datasets:

The evolution of antibiotic resistance is associated with collateral drug phenotypes in *Mycobacterium tuberculosis*

Natalie J.E. Waller[1,2], Chen-Yi Cheung[1], Gregory M. Cook[1,2] and Matthew B. McNeil[1,2#]

1: Department of Microbiology and Immunology, University of Otago, New Zealand.

2: Maurice Wilkins Centre for Molecular Biodiscovery, University of Auckland, New Zealand.

#Corresponding Author

matthew.mcneil@otago.ac.nz

File Name: Supplementary dataset 1

Description: Fold change in MIC between drug resistant and isogenic drug susceptible parent strain for each drug. Fold change values are presented as a log3 number, and are the average of at least 4 biological replicates. Strains are plotted along the X-axis and drug test along the Y-axis. A value of 1 or -1 indicates a 3 fold increase or decrease in MIC. Data is used to plot heatmap in figure 1.

File Name: Supplementary dataset 2

Description: List of mutations identified in each drug-resistant strain when compared to the isogenic drug susceptible parent. Column A denotes drug-resistant strains that were isolated in the same experiment from the same parental culture. Column b denotes strain name of each drug resistant strain, with column c denoting the total number of mutations identified between each drug-resistant strain when compared to the isogenic drug susceptible parent. Column D-G denote the most probable mutation responsible for resistance to the isolating compound, with the amino acid change, nucleotide change and frequency of the most probable mutation across the obtained illumina sequencing reads in columns D, E and F respectively. Column G indicates whether the most probable primary resistance mutation has been observed in drug-resistant clinical isolates, and includes an associated reference. Remaining columns denote additional secondary mutations identified with the amino acid change, nucleotide change and frequency of the mutation in illumina sequencing reads listed.
