# Supplementary material for: The evolution of antibiotic resistance is associated with collateral drug phenotypes in Mycobacterium tuberculosis
Source: Nat Commun. 2023 Mar 18;14:1517. doi: 10.1038/s41467-023-37184-7 (PMC10024696; doi:10.1038/s41467-023-37184-7)
Supplement: Supplementary file 6 — Reporting Summary [file 41467_2023_37184_MOESM6_ESM.pdf]

nature portfolio

Corresponding author(s): Matthew B McNeil

Last updated by author(s): Feb 27, 2023

Reporting Summary

Nature Portfolio wishes to improve the reproducibility of the work that we publish. This form provides structure for consistency and transparency in reporting. For further information on Nature Portfolio policies, see our Editorial Policies and the Editorial Policy Checklist.

Statistics

For all statistical analyses, confirm that the following items are present in the figure legend, table legend, main text, or Methods section.

n/a	Confirmed	
☐	☒	The exact sample size (*n*) for each experimental group/condition, given as a discrete number and unit of measurement
☐	☒	A statement on whether measurements were taken from distinct samples or whether the same sample was measured repeatedly
☐	☒	The statistical test(s) used AND whether they are one- or two-sided<br>*Only common tests should be described solely by name; describe more complex techniques in the Methods section.*
☒	☐	A description of all covariates tested
☐	☒	A description of any assumptions or corrections, such as tests of normality and adjustment for multiple comparisons
☐	☒	A full description of the statistical parameters including central tendency (e.g. means) or other basic estimates (e.g. regression coefficient) AND variation (e.g. standard deviation) or associated estimates of uncertainty (e.g. confidence intervals)
☐	☒	For null hypothesis testing, the test statistic (e.g. *F*, *t*, *r*) with confidence intervals, effect sizes, degrees of freedom and *P* value noted<br>*Give P values as exact values whenever suitable.*
☒	☐	For Bayesian analysis, information on the choice of priors and Markov chain Monte Carlo settings
☒	☐	For hierarchical and complex designs, identification of the appropriate level for tests and full reporting of outcomes
☒	☐	Estimates of effect sizes (e.g. Cohen's *d*, Pearson's *r*), indicating how they were calculated

Our web collection on statistics for biologists contains articles on many of the points above.

Software and code

Policy information about availability of computer code

Data collection	No software was used in data collection
Data analysis	GraphPad Prism V9 was used for the generation of figures and statistical analysis. Geneious Prime® 2020.2.4 for WGS analysis (+BBDuk V1.0 and Bowtie-2 V7.2.2 plugins).

For manuscripts utilizing custom algorithms or software that are central to the research but not yet described in published literature, software must be made available to editors and reviewers. We strongly encourage code deposition in a community repository (e.g. GitHub). See the Nature Portfolio guidelines for submitting code & software for further information.

Data

Policy information about availability of data

All manuscripts must include a data availability statement. This statement should provide the following information, where applicable:

- Accession codes, unique identifiers, or web links for publicly available datasets
- A description of any restrictions on data availability
- For clinical datasets or third party data, please ensure that the statement adheres to our policy

The source data used to generate the figures in this study is available through figshare (https://doi.org/10.6084/m9.figshare.c.6443654.v1). All raw Illumina reads used to generate whole genome sequences are available at NCBI-SRA (BioProject number: PRJNA914416). The H37Rv genome used for mapping of WGS was

obtained from Genbank (accession number: NC_000962 [https://www.ncbi.nlm.nih.gov/nuccore/NC_000962]). Additional data is available from the corresponding author upon request.

Human research participants

Policy information about studies involving human research participants and Sex and Gender in Research.

Reporting on sex and gender	Not Applicable
Population characteristics	Not Applicable
Recruitment	Not Applicable
Ethics oversight	Not Applicable

Note that full information on the approval of the study protocol must also be provided in the manuscript.

Field-specific reporting

Please select the one below that is the best fit for your research. If you are not sure, read the appropriate sections before making your selection.

☒ Life sciences ☐ Behavioural & social sciences ☐ Ecological, evolutionary & environmental sciences

For a reference copy of the document with all sections, see nature.com/documents/nr-reporting-summary-flat.pdf

Life sciences study design

All studies must disclose on these points even when the disclosure is negative.

Sample size	With the exception of MIC dose response curves, data is presented as the average of biological replicates from a representative experiment of at least two independent experiments. MIC dose response curves are presented as data from a single biological replicate from a representative experiment of at least 4 independent experiments that showed a consistent trend.<br><br>The numbers of replicates within each experiment and confirmation through multiple independent experiments is sufficient to show that results seen consistently across replicates are not due to chance.
Data exclusions	When generating MIC curves growth points that were >150% of the no compound control were excluded from analysis only when flanked by values that were >75% of the no compound control
Replication	All results were validated by performing at least 2 replicate experiments. Positive results that showed a difference between parental and mutant strains were validated with at least 3 replicate experiments.
Randomization	Randomization was not relevant to this study. Strains were not treated with randomization. All drug-resistant strains were compared to the drug-susceptible parent strain that was included in each experiment.
Blinding	Investigators were not blinded during data collection or analysis. Data that was collected was not subject to biases that could be introduced by the researcher.

Reporting for specific materials, systems and methods

We require information from authors about some types of materials, experimental systems and methods used in many studies. Here, indicate whether each material, system or method listed is relevant to your study. If you are not sure if a list item applies to your research, read the appropriate section before selecting a response.

Materials & experimental systems

n/a	Involved in the study	
☒	☐	Antibodies
☐	☒	Eukaryotic cell lines
☒	☐	Palaeontology and archaeology
☒	☐	Animals and other organisms
☒	☐	Clinical data
☒	☐	Dual use research of concern

Methods

n/a	Involved in the study	
☒	☐	ChIP-seq
☒	☐	Flow cytometry
☒	☐	MRI-based neuroimaging

Eukaryotic cell lines

Policy information about cell lines and Sex and Gender in Research

Cell line source(s)	THP-1 is a monocyte isolated from peripheral blood from an acute monocytic leukemia patient. THP-1 cell line was obtained from ATCC (Cat# TIB-202).
Authentication	Cell lines were not authenticated
Mycoplasma contamination	Cell lines were not tested for mycoplasma
Commonly misidentified lines (See ICLAC register)	No commonly misidentified cell lines were used in this study
